# Quinoa protein hydrolysates: antioxidant properties and cytoprotection against D-galactose-induced oxidative stress

**DOI:** 10.3389/fnut.2025.1707365

**Published:** 2026-01-13

**Authors:** Cheng Yang, Chao Yang, Yuanyuan Zhou, Jibing Ma, Yuming Wei, Jie Huang

**Affiliations:** 1 Agricultural Product Storage and Processing Research Institute, Gansu Academy of Agricultural Sciences, Lanzhou, Gansu, China; 2 Academy of Animal and Veterinary Sciences, Qinghai University, Xining, China; 3 Animal Husbandry, Pasture and Green Agriculture Institute, Gansu Academy of Agricultural Sciences, Lanzhou, China

**Keywords:** antioxidant, enzymatic hydrolysate, *in vivo*, oxidative damage, quinoa

## Abstract

Quinoa is widely recognized as a high-quality protein source suitable for producing bioactive hydrolysates with significant health-promoting potential. This study compared four proteases (alcalase, trypsin, pepsin, and neutral protease) to identify optimal enzymatic conditions for generating antioxidant quinoa protein hydrolysates (QPHs). Among them, alcalase exhibited the highest degree of hydrolysis (36.15%) and yielded the largest proportion of low-molecular-weight fragments (< 2 kDa, 41.08%). It also produced hydrolysates with the lowest surface hydrophobicity and particle size, reflecting more extensive structural disruption, and demonstrated the highest essential amino acid content (20.72 g/100 g). Functionally, alcalase-derived QPHs showed the strongest DPPH radical scavenging activity (4.30 mg TE/g), the highest reducing power (0.61), and provided the greatest cytoprotection in HepG2 cells (83.43%), significantly enhancing SOD, GPx, and CAT activities while reducing MDA levels by 57.8%. Pepsin hydrolysate exhibited selective enhancement, showing the highest ABTS radical scavenging capacity (7.22 mg TE/g) and the greatest FRAP value (13.26 μmol TE/100 g DW), likely attributable to aromatic-rich fragments generated according to its cleavage specificity. Overall, these findings demonstrate that enzymatically derived QPHs, particularly those produced by alcalase, possess strong antioxidant activity and represent promising natural antioxidant ingredients for functional foods and nutraceutical formulations. The results also offer novel mechanistic insights supporting their application in health-promoting food products.

## 1 Introduction

The demand for valuable protein sources is increasing, driven by global population growth, evolving dietary preferences, and the pursuit of sustainable alternatives to conventional animal-based proteins (1). In this context, quinoa (*Chenopodium quinoa* Willd.) has emerged as a promising plant-derived protein source and a strategically important pseudo-cereal due to its exceptional nutritional composition and remarkable ecological plasticity (2). Native to the Andean region, quinoa thrives across diverse environmental gradients-including high-altitude, drought-prone, and frost-exposed terrains-making it a critical crop for climate-resilient agriculture (3). Its grains contain a well-balanced amino acid profile rich in lysine and methionine, substantial dietary fiber, essential minerals, and a variety of antioxidant phytochemicals (4, 5). Moreover, its gluten-free nature enhances its value for individuals with celiac disease or wheat intolerance. Despite these advantages, quinoa contains saponins located in the pericarp, which impart bitterness and pose challenges to consumer acceptance and large-scale utilization (6, 7). These limitations highlight the need for innovative processing strategies that can enhance quinoa's functional characteristics while mitigating antinutritional factors.

Enzymatic hydrolysis is increasingly recognized as an efficient and environmentally friendly strategy to improve plant protein functionality (8, 9). Proteases can modulate protein conformation and physicochemical attributes-including molecular-weight distribution, hydrophobicity, and interfacial activity-thereby enhancing solubility, emulsification, and processing performance in food systems (10–13). Beyond improving functionality, enzymatic proteolysis generates bioactive substances with beneficial physiological activities. Of particular relevance are antioxidant capacity, which exert their effects through free-radical scavenging, metal chelation, and inhibition of lipid peroxidation (14–17). Previous studies on legume proteins—such as black bean (13) and chickpea (17)—have underscored that enzyme specificity plays a decisive role in determining the structure and bioactivity of generated hydrolysates.

Notably, different proteases produce products with distinct molecular characteristics due to their unique cleavage specificities (18, 19). Alcalase, a broad-spectrum serine endopeptidase, preferentially cleaves hydrophobic regions, generating low-molecular-weight fragments enriched in hydrophobic and aromatic residues-structural features strongly associated with enhanced antioxidant potential (18). Trypsin targets Lys and Arg residues, yielding positively charged fragments that may exhibit superior metal-chelating ability and radical-quenching efficiency (20, 21). Pepsin, active under acidic conditions, favors cleavage at aromatic residues, often releasing fragments with strong electron-donating capacity (22). Neutral protease produces moderately sized fragments through non-specific cleavage under near-neutral conditions, typically resulting in balanced hydrophobicity and charge distribution (19). These enzyme-dependent differences in cleavage specificity fundamentally shape the resulting products' charge profiles, hydrophobicity levels, and consequently their antioxidant behaviors-mechanistic relationships that remain underexplored in quinoa proteins (23).

However, despite quinoa's growing prominence, systematic investigations into how these protease-specific mechanisms influence the structural features, antioxidant activities, and cellular protective effects of QPHs remain limited. The objective of this study was to comprehensively investigate the structural attributes and antioxidant activities of QPHs produced using four different proteases. Structural characterization was performed through analysis of amino acid composition, particle size distribution, and zeta potential. Antioxidant capacities were evaluated using multiple *in vitro* assays, including Ferric Ion-Reducing Antioxidant Power (FRAP), reducing power assay, and DPPH and ABTS radical scavenging activities. Furthermore, the cytoprotective effects of QPHs against D-galactose-induced oxidative stress were assessed in HepG2 cells. This work provides a mechanistic foundation for the development of quinoa-derived antioxidant peptides as promising functional ingredients for food and health-related applications.

## 2 Materials and methods

### 2.1 Material

The quinoa variety Longli No. 1 was obtained from the Gansu Academy of Agricultural Sciences. To ensure batch consistency, all quinoa grains used in this study were sourced from a single seed lot and cultivated under uniform field management conditions during the same growing season. After harvest, the grains underwent identical postharvest handling procedures, including cleaning, drying, and storage under controlled conditions. These steps were implemented to ensure that all materials were genetically homogeneous and exhibited minimal compositional variation. The grains were then ground and passed through a 60-mesh sieve to obtain quinoa powder, which was packaged in airtight valve bags and stored at −20 °C until use. All reagents used in this study were of analytical grade.

### 2.2 Extraction of QP

Briefly, quinoa was initially defatted using petroleum ether (1:5, w/v) in a Soxhlet extractor for 6 h. The defatted quinoa was then mixed with distilled water (1:10, w/v), and the pH was adjusted to 10.0 using 1 mol/L NaOH. The resulting mixture was continuously stirred in a water bath at 30 °C for 3 h, followed by centrifugation at 5,000 × g for 30 min. This extraction procedure was repeated three times. The protein was then obtained by collecting the supernatants, which were precipitated at pH 4.5 with 1 mol/L HCl. The precipitate was incubated at 4 °C for 24 h before being collected by centrifugation at 5,000 × g for 30 min and finally lyophilized for further analysis. Given that quinoa proteins contain 11S globulins and 2S albumins with distinct solubility profiles, precipitation at pH 4.5 may selectively enrich globulins while partially depleting albumins.

### 2.3 Preparation of QPHs

As outlined by Du et al. (24) with modifications, QPHs were prepared using alcalase (54 °C, pH 8.5), trypsin (optimal conditions: 41 °C, pH 8.5), neutral protease (52 °C, pH 6.5), and pepsin (40 °C, pH 1.5). During hydrolysis, the pH of the enzymatic dispersion was adjusted by adding NaOH (2 mol/L) dropwise, with the mixture being stirred using an overhead agitator (DLAB Scientific Co. Ltd., Beijing, China). After hydrolysis, the reaction mixture was heated at 100 °C for 20 min to deactivate the enzymes, then cooled to 25 °C and centrifuged at 9,000 × g for 10 min to separate the supernatant. The supernatant was freeze-dried to obtain the QPH. The hydrolysates were labeled as A-QPH, T-QPH, N-QPH, and W-QPH, with protein recoveries of 94.51, 93.74, 92.48, and 91.24%, respectively.

### 2.4 Selecting the appropriate enzyme

#### 2.4.1 Determination of degree of hydrolysis (DH)

The DH of the enzymatic hydrolysates was determined using the o-phthaldialdehyde (OPA) colorimetric method established by Nielsen et al. (25). Briefly, the hydrolysate samples were dissolved in 0.1 mol/L phosphate buffer (pH 7.0) to obtain a final concentration of 2 mg/ml. The prepared solutions were thoroughly mixed with the OPA reagent at the molecular level by vortexing. The reaction mixture was then incubated under light-protected conditions for 2 min. Subsequently, the absorbance was measured at 340 nm using a UV-Vis spectrophotometer. The DH was calculated according to Equation 1.


$$\begin{array}{l} \text{DH}\left(\%\right)=\frac{\text{h}}{{\text{h}}_{\text{tot}}}\times 100\% \end{array}$$ (1)

In the equation, h represents the number of peptide bonds cleaved during hydrolysis, while h_tot_ denotes the total number of peptide bonds present in the protein substrate.

#### 2.4.2 Determination of molecular weight distribution

According to Xu et al. (26), the MWD of the hydrolysates was determined using a HPLC system (U3000, Thermo Fisher Scientific, USA) equipped with a size-exclusion column (BIOSEP SEC-S2000, 5 μm, 300 × 7.8 mm; Phenomenex, CA, USA). The mobile phase comprised acetonitrile and ultrapure water (45:55, v/v) containing 0.1% trifluoroacetic acid, and isocratic elution was carried out at a flow rate of 0.5 ml/min with a 20 μl injection volume. Chromatographic profiles of the hydrolysates were compared with those of a standard mixture of compounds with known molecular weights, and detection was performed at 214 nm. The standards included Gly-Gly-Gly (189 Da), Gly-Gly-Tyr-Arg (451 Da), bacitracin (1,423 Da), and aprotinin (6,511 Da).

#### 2.4.3 Surface hydrophobicity

Chelh et al. (27) adopted the bromophenol blue (BPB) binding assay to evaluate the surface hydrophobicity of proteins. Initially, a quinoa protein suspension (5 mg/ml in PBS, pH 7.0) was combined with 200 μl of BPB solution (1 mg/ml), allowing sufficient interaction for binding. A blank control was prepared by mixing 200 μl of BPB with 1 ml of PBS. The mixture was stirred for 10 min and subsequently centrifuged at 2,000 × g for 15 min. The absorbance of the resulting supernatant was then recorded at 595 nm.


$$\begin{array}{l} \text{BPB bound}\left(\text{μg}\right)=200\;\mu \text{g}\times^{\left(A_{\text{control}}-A_{\text{sample}}\right)}{/}_{A_{\text{control}}} \end{array}$$ (2)

#### 2.4.4 Particle size and polydispersity index (PDI)

Deionized water was used to dissolve the sample, followed by the assessment of particle size and distribution in the sample at 25 °C using a laser diffraction analyzer (Mastersizer 2000, Malvern, UK) (28).

### 2.5 Amino acid (AA) composition

The AA analysis was conducted following the procedure described by Machado et al. (29). Briefly, 0.1 g of lyophilized QPHs powder was dissolved in 10 ml of 6 M HCl in a hydrolysis tube, which was subsequently purged with nitrogen for 5 min. Acid hydrolysis was then performed in vacuum-sealed tubes at 110 °C for 22 h. Tryptophan was quantified separately after alkaline hydrolysis under identical conditions. Following hydrolysis, the samples were cooled and the hydrolysate was brought to a final volume of 25 ml. A 2-ml aliquot of the digest was collected and evaporated at 60 °C for 24 h. The residue was reconstituted in 2 ml of ultrapure water and dried again; this dissolution–drying cycle was repeated three times. The resulting material was dissolved and diluted to 5 ml, after which a 2-ml portion was filtered through a 0.22-μm aqueous membrane for analysis. Amino acid separation was performed using an automated AA analyzer (LA8080; Hitachi Co., Tokyo, Japan) equipped with a protein hydrolysate analysis column, operating at a flow rate of 0.40 ml/min. Individual amino acids were identified and quantified by comparing sample chromatographic profiles with those of standard AA mixtures.

### 2.6 Antioxidant capacity

#### 2.6.1 DPPH radical-scavenging activity

The DPPH radical-scavenging activity was determined according to the method described by Repo-Carrasco-Valencia et al. (30), with slight modifications. Briefly, a DPPH working solution (0.1 mmol/L) was prepared in methanol and freshly prepared before use. An aliquot of 1.0 ml of the hydrolysate solution was mixed with 4.5 ml of DPPH working solution, vortexed thoroughly, and incubated in the dark at room temperature for 30 min. Methanol was used as the blank. Absorbance was measured at 517 nm using a UV–Vis spectrophotometer. Vitamin C was included as a positive control to verify assay responsiveness. Antioxidant activity was quantified using a Trolox calibration curve and expressed as μmol Trolox equivalents (TE) per 100 g dry extract.

#### 2.6.2 ABTS free radical-scavenging activity

The ABTS radical-scavenging activity was evaluated following the method reported by Shao et al. (31). The ABTS radical cation (ABTS•^+^) working solution was prepared by mixing 7.0 mmol/L ABTS stock solution with 2.45 mmol/L potassium persulfate and allowing the mixture to react in the dark at room temperature for 12–16 h. Prior to analysis, the ABTS•^+^ solution was diluted with methanol to obtain an absorbance of 0.70 ± 0.02 at 734 nm. For the assay, 200 μl of the hydrolysate solution was added to 4.0 ml of ABTS working solution, vortexed, and incubated in the dark for 30 min. Methanol served as the blank. Absorbance was measured at 734 nm. Vitamin C was used as a positive control. Results were calculated based on a Trolox standard curve and expressed as μmol TE per 100 g dry weight (DW).

#### 2.6.3 Ferric ion-reducing antioxidant power (FRAP)

The ferric ion-reducing antioxidant power (FRAP) assay was conducted according to the method of Pang et al. (32). The FRAP working solution was freshly prepared by mixing acetate buffer (300 mmol/L, pH 3.6), TPTZ solution (10 mmol/L in 40 mmol/L HCl), and FeCl3·6H_2_O solution (20 mmol/L) at a volume ratio of 10:1:1 (v/v/v). For the assay, 1.0 ml of the sample solution was mixed with 4.5 ml of FRAP working solution, gently vortexed, and incubated in the dark at room temperature for 30 min. Methanol was used as the blank. Absorbance was measured at 593 nm. All measurements were performed in triplicate. FRAP values were quantified using a Trolox calibration curve and expressed as μmol TE per 100 g dry weight (DW).

#### 2.6.4 Determination of reducing power

Reducing power was measured based on the method of You et al. (33). The hydrolysate solution, phosphate buffer (pH 6.6), and potassium ferricyanide were mixed (1:2.5:2.5), incubated at 50 °C for 20 min, and treated with 10% trichloroacetic acid. After centrifugation (3,500 × g, 10 min), the supernatant was mixed with 0.1% ferric chloride solution (5:1, v/v), incubated for 10 min in the dark, and absorbance was read at 700 nm. Vitamin C was used as the positive control to verify method performance. Higher absorbance indicated stronger reducing power.

### 2.7 Evaluation of the antioxidant activity of QPHs in a cellular model

#### 2.7.1 Determination of cytotoxicity

The cytotoxicity of QPHs was evaluated using the MTT assay as described by Wen et al. (34). Briefly, HepG2 cells in the logarithmic growth phase were seeded into 96-well plates at a density of 5 × 104 cells/well. After incubation under standard conditions (37 °C, 5% CO_2_) for 24 h to allow cell adherence, the culture medium was removed and replaced with fresh medium containing different concentrations of QPHs (0–200 μg/ml). The cells were exposed to the treatments for 12 h. Subsequently, the supernatant was discarded, and 100 μl of MTT working solution (1.0 mg/ml) was added to each well, followed by incubation in the dark for 4 h. After incubation, the MTT solution was removed, and 150 μl of dimethyl sulfoxide was added to dissolve the blue formazan crystals. The absorbance was measured at 570 nm using a microplate reader (*n* = 6, CV < 4.5%). Cell viability was calculated according to Equation 3.


$$\text{Cell Viability }\left(\%\right)\text{=}\left(\frac{{\text{A}}_{\text{blank}}{\text{-A}}_{\text{sample}}}{{\text{A}}_{\text{blank}}}\right)\times \text{100}\%$$ (3)

#### 2.7.2 The protective effects of QPHs against cellular oxidative damage

The protective effect of QPHs against D-galactose-induced oxidative damage in HepG2 cells was evaluated using the MTT assay, following the method of Wen et al. (34). Briefly, HepG2 cells were seeded in 96-well plates at a density of 5 × 104 cells/well and incubated under standard conditions for 24 h to allow adherence. Cells were then pretreated with QPHs (100 μg/ml) for 4 h, followed by exposure to D-galactose (400 μmol/L) for 6 h to induce oxidative damage. After the treatment, the cells were gently washed with PBS and cultured in fresh medium for an additional 6 h to allow recovery. Subsequently, 100 μl of MTT solution (1.0 mg/ml) was added to each well, and the cells were incubated in the dark for 4 h. The supernatant was then removed, and 150 μl of dimethyl sulfoxide was added to dissolve the blue formazan crystals. The absorbance was measured at 570 nm using a microplate reader. Cell viability was calculated according to Equation 3.

#### 2.7.3 Determination of oxidative stress markers in the cellular model

The antioxidant effects of QPHs were further evaluated using a D-galactose-induced oxidative stress model in HepG2 cells, as described by Wen et al. (34). Briefly, HepG2 cells in the logarithmic growth phase were seeded into 6-well plates at a density of 2 × 10^5^ cells/well and cultured for 24 h to allow adherence. After establishing the damage model, the cells were collected and lysed with 200 μl of pre-chilled lysis buffer per well, followed by incubation on ice for 30 min. The lysates were centrifuged at 12,000 × g for 10 min at 4 °C, and the supernatant was collected for analysis. The protein concentration of each sample was determined using the bicinchoninic acid method to ensure consistency. Subsequently, the levels of MDA, SOD, CAT, and GPx levels were measured using commercial assay kits according to the manufacturers' instructions (Nanjing Jiancheng Biotechnology, Nanjing, China).

### 2.8 Statistical analysis

The experiment was conducted in triplicate, and all data were expressed as mean ± SD. Statistical analyses were performed using one-way analysis of variance (ANOVA) in SPSS. Prior to ANOVA, the homogeneity of variances was assessed using Levene's test. When significant differences were detected (*P* < 0.05), *post-hoc* comparisons were carried out using either the least significant difference (LSD) test or Duncan's multiple range test, depending on the data characteristics. Letter-based grouping was automatically assigned by SPSS to indicate statistically distinct subsets. Graphs and tables illustrating the results were generated using Origin 8.0.

## 3 Results

### 3.1 Structural features of hydrolyzed quinoa proteins

#### 3.1.1 Degree of hydrolysis

**Figure 1:**
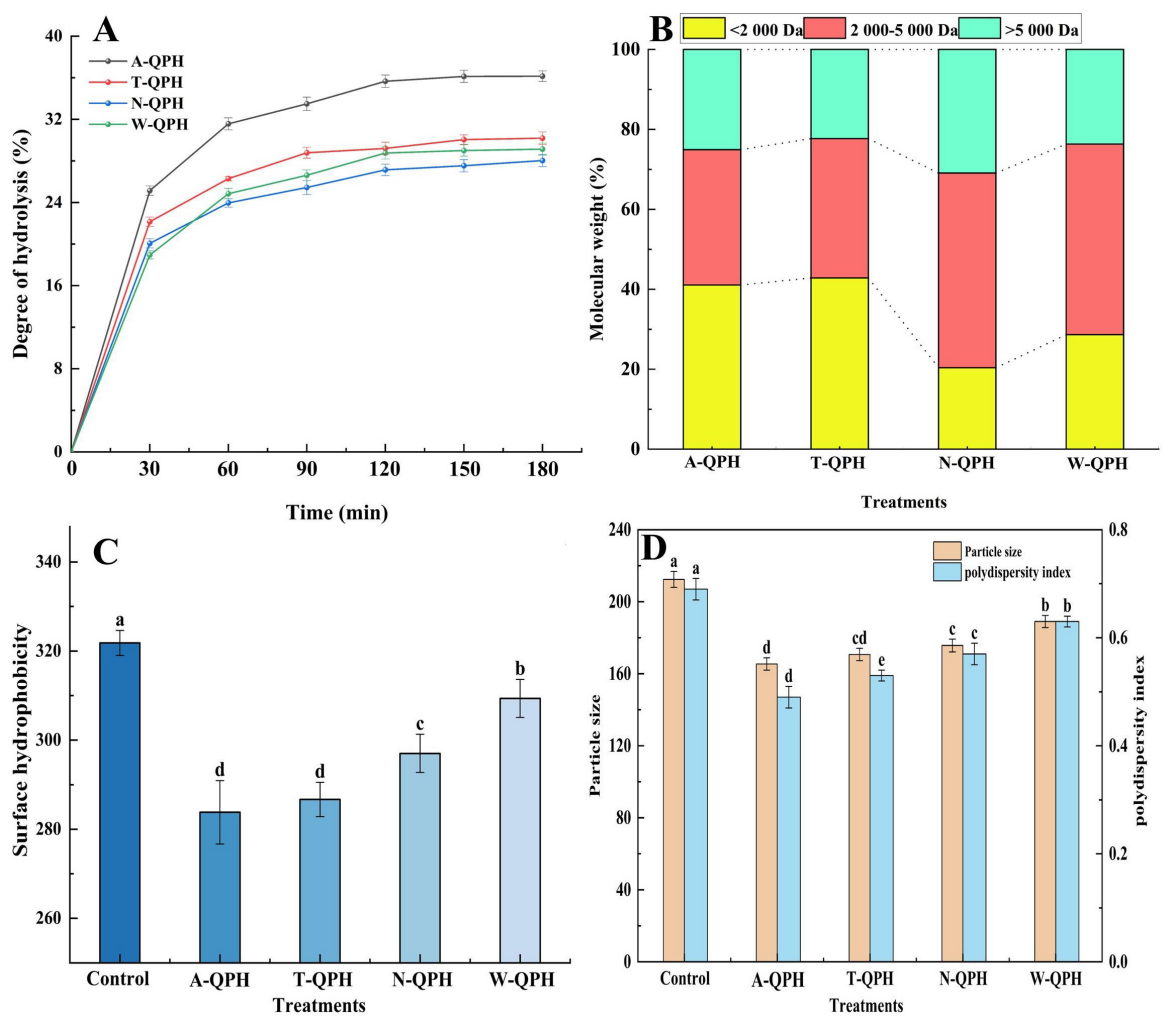
Effects of different protease treatments on the physicochemical properties of quinoa protein hydrolysates. **(A)** Degree of hydrolysis (DH); **(B)** molecular weight distribution; **(C)** surface hydrophobicity; **(D)** average particle size and polydispersity index (PDI). Values are expressed as means ± standard deviations (*n* = 3). Different lowercase letters indicate statistically significant differences among enzyme treatments (*P* < 0.05).

As shown in Figure 1A, the DH of QPHs increased steadily during the first 180 min of enzymatic treatment for all enzymes tested and gradually approached a plateau thereafter. Among the four enzymes, alcalase produced the highest DH at 180 min (36.15%), which was significantly greater than that of trypsin (30.19%), pepsin (29.13%), and neutral protease (28.04%) (*P* < 0.05). These results demonstrate the superior hydrolytic efficiency of alcalase in quinoa protein hydrolysis.

#### 3.1.2 Molecular weight distribution

The molecular weight distribution of QPHs is shown in Figure 1B. Both trypsin and alcalase hydrolysates contained a higher proportion of low-molecular-weight QPHs (< 2 kDa), accounting for 42.85 and 41.08%, respectively, indicating their strong capacity to generate small QPHs. In contrast, pepsin and neutral protease hydrolysates predominantly produced QPHs in the 2–5 kDa range, comprising 48.7 and 47.69%, respectively, whereas the proportions in the trypsin and alcalase groups were comparatively lower. Regarding high-molecular-weight fractions (>5 kDa), trypsin and alcalase hydrolysates accounted for 22.3 and 25.06%, respectively, while pepsin and neutral protease hydrolysates exhibited higher levels, reaching 30.93 and 23.67%, respectively.

#### 3.1.3 Surface hydrophobicity

As shown in Figure 1C, different enzymatic treatments significantly influenced the surface hydrophobicity of quinoa protein hydrolysates (*P* < 0.05). The surface hydrophobicity of native quinoa protein was 321.82. Following enzymatic hydrolysis, the alcalase-treated group exhibited the lowest surface hydrophobicity (283.81), which was significantly lower than that of the other enzyme-treated groups (*P* < 0.05). The trypsin-treated group also showed a significant reduction in surface hydrophobicity (286.69) compared to the native protein (*P* < 0.05). In contrast, the neutral protease- and pepsin-treated groups displayed surface hydrophobicity values of 297.01 and 309.37, respectively, slightly lower than the native protein.

#### 3.1.4 Particle size and PDI

As shown in Figure 1D, the particle size and PDI of quinoa protein were 212.38 nm and 0.69, respectively. The alcalase-treated group exhibited the smallest particle size (165.45 nm) and a significantly reduced PDI (0.49) compared to the other groups (*P* < 0.05). The trypsin-treated group displayed a particle size of 170.69 nm and a PDI of 0.53, both significantly lower than those of the native protein and the pepsin-treated group (*P* < 0.05). The neutral protease-treated group showed a particle size of 175.69 nm and a PDI of 0.57, whereas the pepsin-treated group had values of 189.03 nm and 0.63, which were significantly higher than those of the other enzyme-treated groups (*P* < 0.05).

### 3.2 Amino acid composition

The amino acid composition of QPHs obtained via different enzymatic hydrolysis treatments is summarized in Table 1. The total content of EAAs varied minimally among the groups, with the alcalase-treated samples showing slightly higher EAA levels (20.72 g/100 g), significantly exceeding those of the trypsin (20.47 g/100 g), pepsin (20.36 g/100 g), and neutral protease (20.26 g/100 g) groups (*P* < 0.05). Lys levels remained comparable across treatments, ranging from 3.08 to 3.18 g/100 g, whereas Met was significantly enriched in the alcalase-treated hydrolysates (1.86 g/100 g). Conversely, a slight reduction in Ser was observed after neutral protease hydrolysis. For NEAAs, alcalase also yielded significantly higher Glu and Asp contents (9.67 and 6.24 g/100 g, respectively; *P* < 0.05), suggesting preferential liberation of acidic residues.

**Table 1:** Effect of different protease treatments on the amino acid composition of quinoa protein hydrolysates.

**Amino acids**	**Content (g/100 g protein)**				

	**Control**	**A-QPH**	**T-QPH**	**N-QPH**	**W-QPH**
**Essential amino acids**					
Leu	4.23	4.21	4.17	4.07	4.09
Ile	2.76	2.73	2.71	2.84	2.79
Lys	3.14	3.08	3.18	3.11	3.14
Met	1.68	1.86	1.74	1.77	1.7
Phe	2.84	2.79	2.75	2.83	2.85
Thr	2.08	2.34	2.29	2.17	2.15
Val	2.57	2.71	2.64	2.58	2.59
Try	0.95	1.00	0.99	0.99	0.95
**Non-essential amino acids**					
His	1.86	1.85	1.83	1.87	1.89
Tyr	1.68	1.9	1.84	1.66	1.73
Cys	0.86	0.94	0.9	0.92	0.85
Ala	2.48	2.46	2.46	2.5	2.45
Gly	3.57	3.47	3.45	3.51	3.55
Pro	4.21	4.14	4.09	4.25	4.23
Ser	3.14	3.00	3.02	2.96	3.15
Glu	9.05	9.67	9.53	9.62	9.29
Asp	5.84	6.24	6.28	6.35	5.97
Arg	5.17	5.04	5.02	5.03	5.02
EAA	20.25	20.72	20.47	20.36	20.26
NEAA	37.86	38.71	38.42	38.67	38.13
TAA	58.11	59.43	58.89	59.03	58.39
HAA	24.34	24.37	24.01	24.35	24.25
HLAA	7.76	8.18	8.05	7.71	7.88

HAA: Ala, Ile, Leu, Met, Phe, Val, Gly, Pro; HLAA: Ser, Thr, Cys, Tyr.

EAA, Essential amino acid; NEAA, non-essential amino acid; TAA, total amino acid; HAA, hydrophobic amino acids; HLAA, hydrophilic amino acids.

### 3.3 Antioxidant activity of QPHs

**Figure 2:**
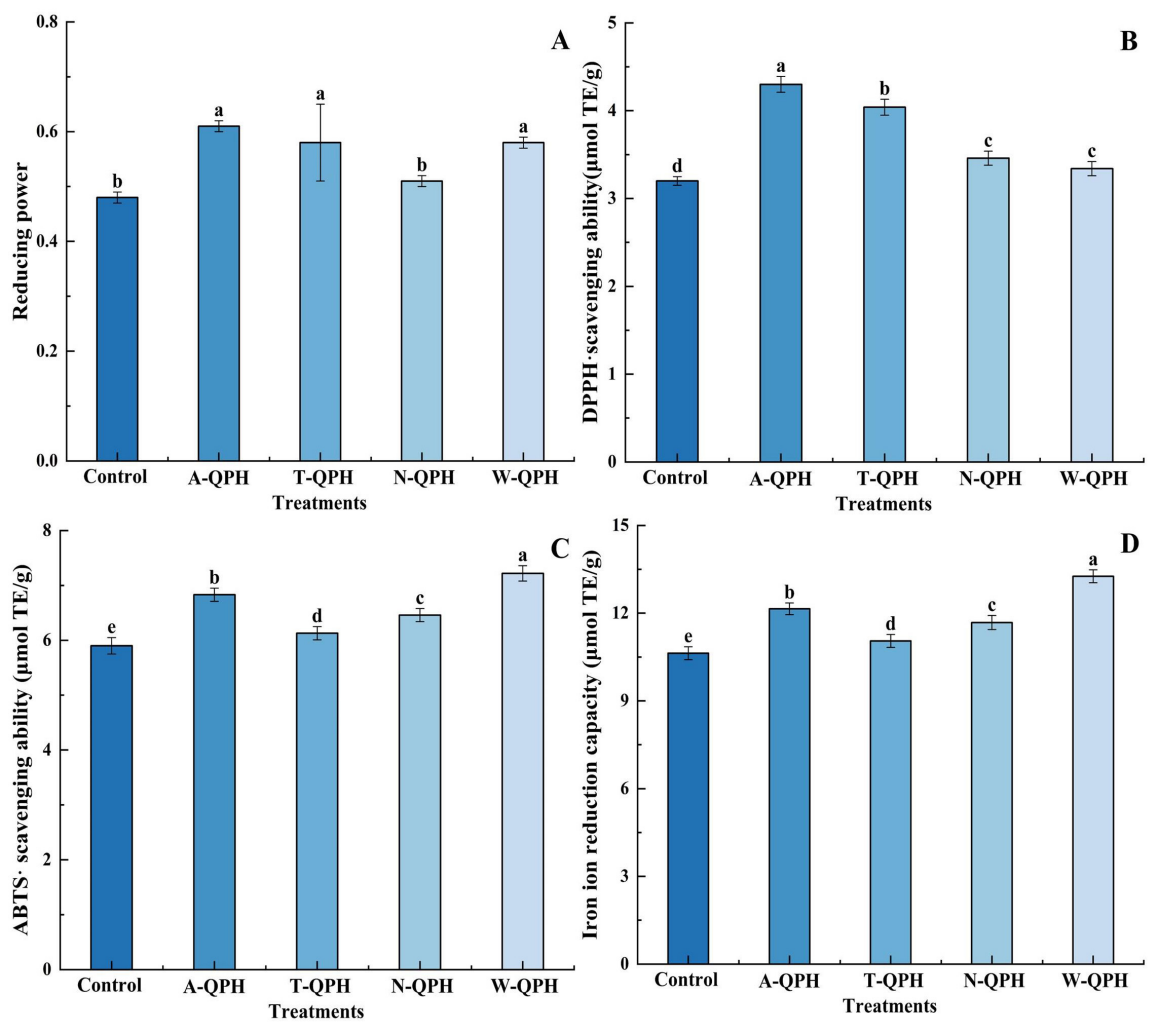
Effects of different protease treatments on the antioxidant activities of quinoa protein hydrolysates *in vitro*. **(A)** Reducing power; **(B)** DPPH radical scavenging activity; **(C)** ABTS radical scavenging activity; **(D)** ferric ion reducing antioxidant power (FRAP). Values are means ± standard deviations (*n* = 3). Different lowercase letters indicate significant differences among enzyme treatments (*P* < 0.05).

As shown in Figure 2, the antioxidant properties of QPHs were markedly influenced by the type of protease employed during hydrolysis. Among the tested groups, hydrolysates treated with alcalase, pepsin, and trypsin exhibited significantly higher reducing power, with absorbance values of 0.61, 0.58, and 0.58, respectively, compared to the neutral protease-treated group (0.51) (*P* < 0.05). Enzyme-specific antioxidant capacities were further distinguished using radical scavenging assays. The DPPH radical scavenging activity was highest in the alcalase group, reaching 4.30 mg TE/g, whereas the pepsin-treated hydrolysate demonstrated superior ABTS radical scavenging activity, achieving 7.22 mg TE/g. Both values were significantly higher than those observed in the other enzyme treatments (*P* < 0.05). Furthermore, the pepsin hydrolysate displayed the highest FRAP value, measured as 13.26 μmol TE/100 g DW, compared with 12.15 μmol TE/100 g DW in the alcalase group. This improvement suggests that pepsin hydrolysis may promote the exposure of specific metal-binding sites, thereby enhancing redox potential. Collectively, these results indicate that alcalase confers the greatest overall improvement in antioxidant activity, whereas pepsin selectively enhances free radical scavenging and metal ion reduction, highlighting the critical role of enzyme specificity in modulating the functional properties of QPHs.

### 3.4 Antioxidant activity of QPHs in the cellular model

#### 3.4.1 Cytotoxicity of quinoa protein hydrolysates

**Figure 3:**
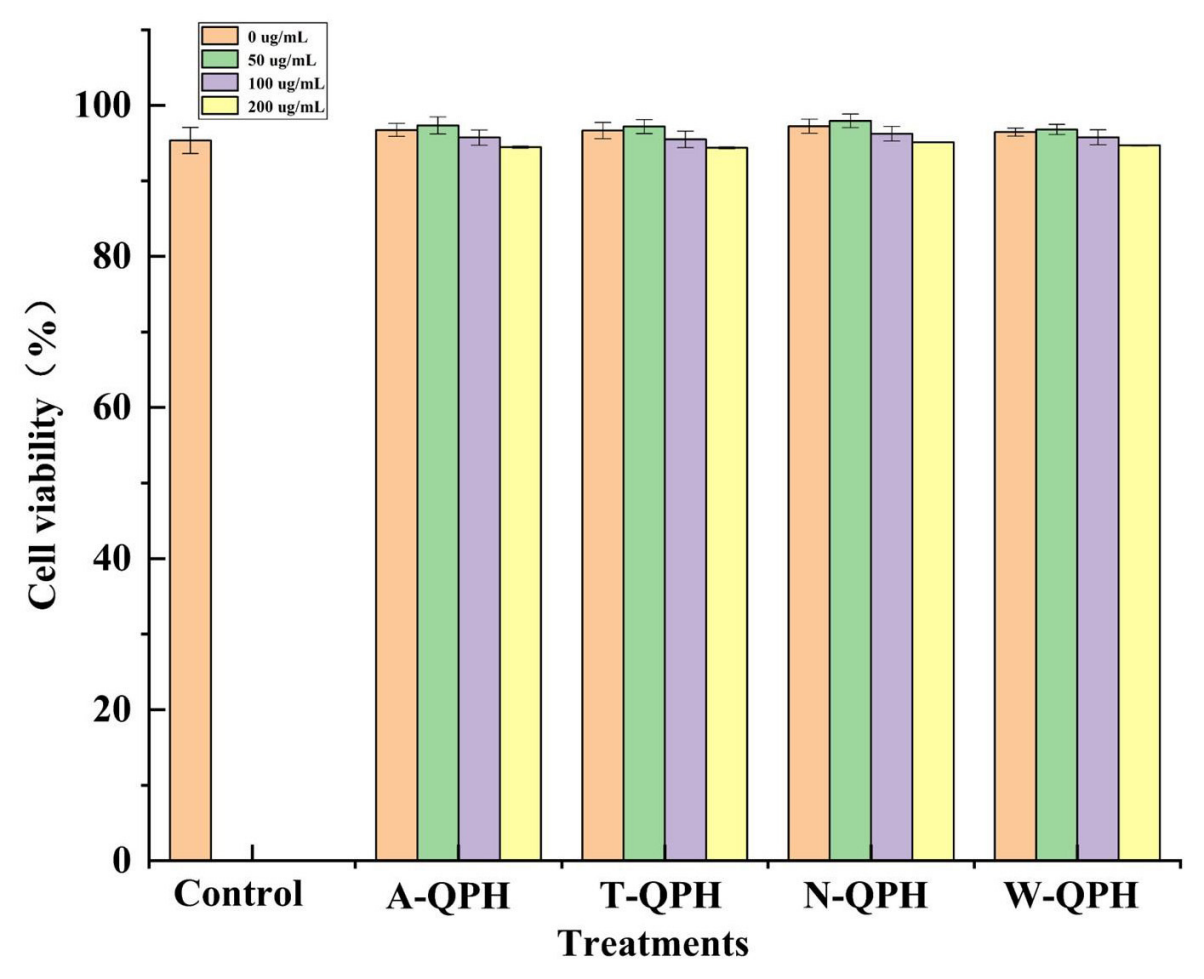
Effects of different protease treatments on the cytotoxicity of quinoa protein hydrolysates in cultured cells. Values are expressed as means ± standard deviations (*n* = 3).

The cytotoxicity of QPHs prepared with different enzymes was assessed in HepG2 cells, as shown in Figure 3. Increasing QPH concentrations (50, 100, and 200 μg/ml) caused a slight decrease in cell viability; however, overall viability remained above 95%, indicating low cytotoxicity. Similar trends were observed across all enzymatic hydrolysate groups-including trypsin, alcalase, pepsin, and neutral protease-with no significant dose-dependent effects. These results suggest that QPHs possess good biocompatibility and low toxicity within the tested concentration range. Consequently, a QPH concentration of 100 μg/ml and its corresponding ultrafiltration fractions were selected for subsequent experiments.

#### 3.4.2 Effects of QPHs on oxidative damage in HepG2 cells

**Figure 4:**
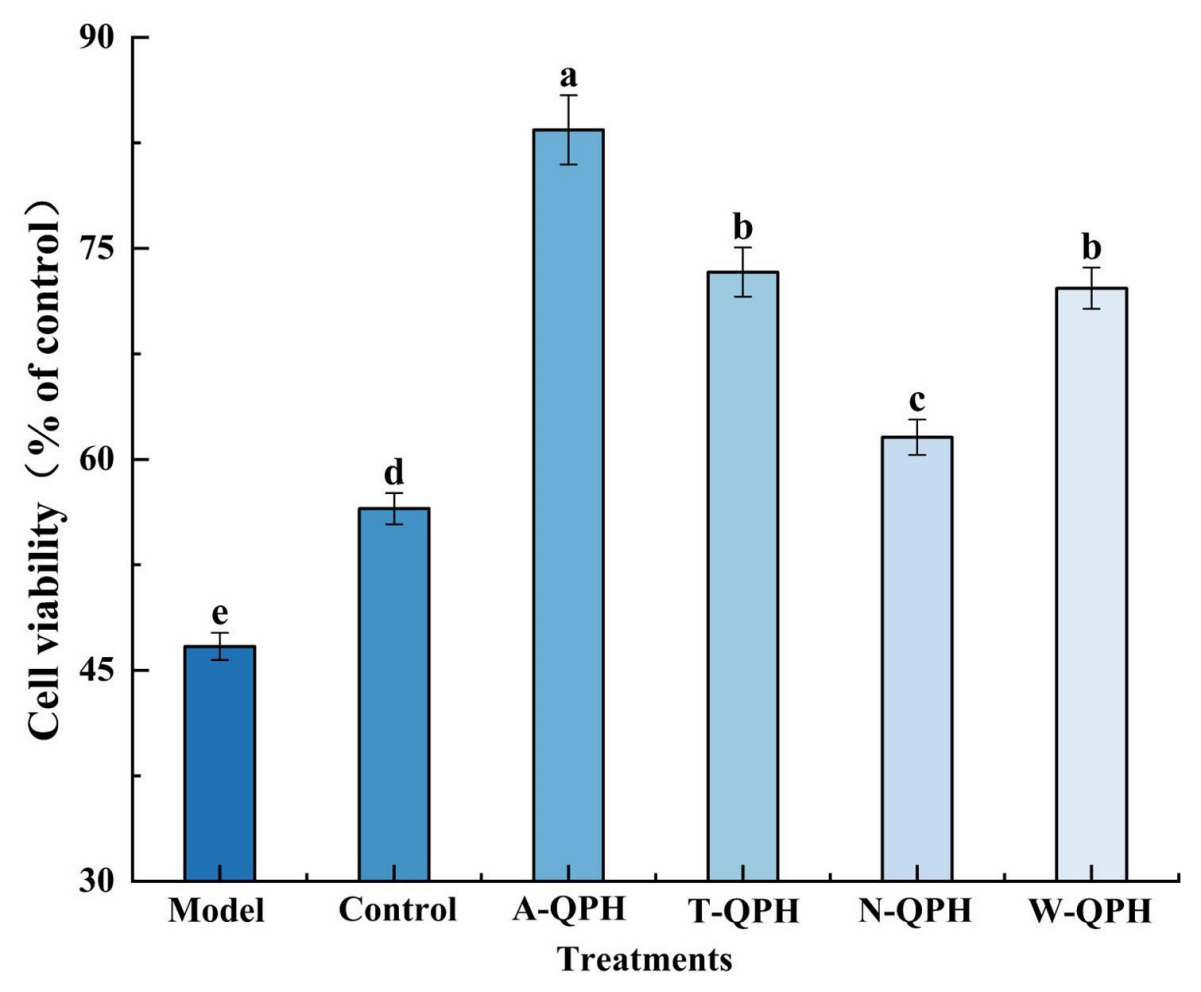
Protective effects of quinoa protein hydrolysates obtained using different proteases against oxidative damage in cultured cells. Values are means ± standard deviations (*n* = 3). Different lowercase letters indicate statistically significant differences among enzyme treatments (*P* < 0.05).

The protective effects of various treatments on oxidative damage in HepG2 cells are presented in Figure 4. Compared with the model group (46.7%), treatment with native quinoa protein significantly enhanced the cell protection rate to 56.5% (*P* < 0.05). Among the hydrolysates, the A-QPH exhibited the strongest protective effect, with a cell protection rate of 83.43%, followed by the T-QPH and W-QPH groups, which reached 73.32 and 72.17%, respectively. The N-QPH showed a moderate protective effect (61.58%), all significantly higher than the model group (*P* < 0.05). Relative to the model group, the protective effects of the alcalase, trypsin, pepsin, and neutral protease groups increased by 78.6, 57.0, 54.5, and 31.9%, respectively (*P* < 0.05). These results indicate that hydrolysates produced by alcalase exert the most pronounced protective effect against D-galactose-induced oxidative damage in HepG2 cells.

#### 3.4.3 Effects of QPHs on antioxidant defense in D-galactose-induced HepG2 cells

**Figure 5:**
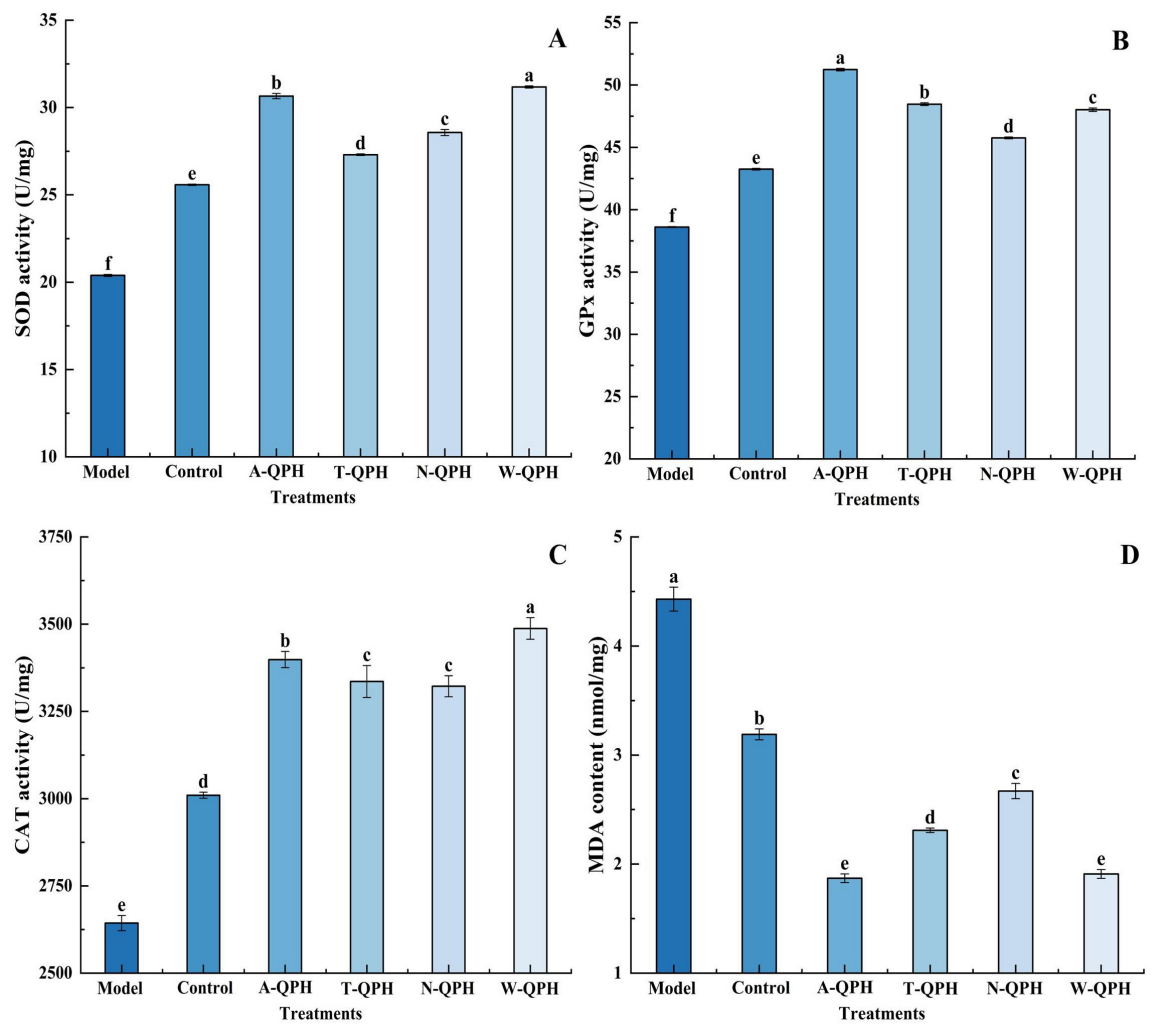
Effects of different protease treatments on intracellular antioxidant enzyme activities and lipid peroxidation levels in cells. **(A)** Superoxide dismutase (SOD) activity; **(B)** glutathione peroxidase (GPx) activity; **(C)** catalase (CAT) activity; **(D)** malondialdehyde (MDA) content. Values are expressed as means ± standard deviations (*n* = 3). Different lowercase letters indicate statistically significant differences among enzyme treatments (*P* < 0.05).

The effects of QPHs on intracellular antioxidant enzyme systems in D-galactose-induced HepG2 cells are presented in Figure 5. Among all treatments, the alcalase hydrolysate exhibited the most comprehensive antioxidant activity. Specifically, SOD activity reached 30.66 U/mg protein, representing a 50.3% increase compared with the model group (20.39 U/mg) (*P* < 0.05). GPx activity was significantly elevated to 51.24 U/mg, a 32.7% improvement over the model group (38.61 U/mg), and CAT activity reached 3,398.76 U/mg, which was significantly higher than that of the trypsin-treated group (*P* < 0.05). Interestingly, the pepsin hydrolysate showed comparable CAT activity (3,487.72 U/mg) and MDA inhibition (1.91 nmol/mg) to the alcalase group, with no significant difference (*P* < 0.05). Moreover, it significantly enhanced SOD and GPx activities by 52.7 and 24.4%, respectively, relative to the model group, indicating a selective regulatory effect. The neutral protease hydrolysate exhibited moderate antioxidant capacity, with SOD, GPx, and CAT activities of 28.57, 45.76, and 3,322.28 U/mg, respectively. In contrast, the trypsin hydrolysate displayed relatively weak antioxidant effects, as evidenced by a significantly higher MDA content (2.31 nmol/mg) compared with the alcalase group (1.87 nmol/mg) (*P* < 0.05). Overall, these results suggest that the alcalase hydrolysate confers superior systemic antioxidant defense by synergistically activating the SOD-GPx-CAT enzyme network and effectively suppressing lipid peroxidation, achieving a 57.8% reduction in MDA levels.

## 4 Discussion

### 4.1 Effects of protease types on the structure of hydrolysates

The quinoa protein substrate employed in this study was obtained via isoelectric precipitation at pH 4.5, a process that preferentially enriches globulin fractions (35). Consequently, the hydrolysis behavior and structural transformations discussed below primarily reflect the response of this globulin-enriched protein fraction, rather than the entire native quinoa grain proteome, and this compositional context should be considered when interpreting enzyme-specific effects. Enzymatic hydrolysis cleaves fragments within proteins, transforming their tightly folded structures into more relaxed conformations (36). The functionality of protein hydrolysates is commonly evaluated using the DH, which reflects the proportion of cleaved peptide bonds (37). In this study, all enzymatic treatments significantly increased the DH of quinoa protein, with alcalase exhibiting the highest hydrolytic efficiency. Similar trends reported in mung bean protein further demonstrate the strong catalytic performance of alcalase relative to other proteases (38). Higher DH values typically correspond to greater fragmentation and improved functional properties, as smaller peptides diffuse more efficiently and interact more readily with biomolecules (39).

Beyond DH differences, the distinct catalytic mechanisms of alcalase, trypsin, pepsin, and neutral protease exert predictable influences on peptide physicochemical characteristics. Alcalase—an alkaline serine endopeptidase—preferentially cleaves at hydrophobic residues, often generating low-molecular-weight peptides (40). Trypsin specifically targets Lys and Arg sites, yielding positively charged peptides, while pepsin favors aromatic residues under acidic conditions. Neutral protease, with broader but milder substrate specificity, produces peptides of moderate hydrophobicity and charge density. These mechanistic distinctions provide a plausible qualitative framework for interpreting the observed differences in molecular-weight distribution, surface hydrophobicity, and particle size among the hydrolysates, although direct peptide-sequence evidence is not available in the present study. The molecular-weight distribution patterns identified in this study-more low-molecular-weight fragments in alcalase and trypsin hydrolysates, and more medium-sized fragments in pepsin and neutral protease hydrolysates-are consistent with these established cleavage preferences (34).

The physicochemical changes observed also align with this qualitative framework. Reductions in surface hydrophobicity following hydrolysis, particularly in alcalase- and trypsin-treated samples, suggest conformational rearrangements and increased exposure of polar groups (41, 42). Decreases in particle size and PDI further support the formation of smaller, more uniformly distributed peptide populations with increased mobility and solubility. Collectively, these structural features provide a coherent basis for interpreting functional differences among enzyme treatments, while acknowledging that peptide-level characterization will be required to confirm specific structure–activity relationships.

### 4.2 Effects of protease types on the antioxidant capacity of hydrolysates

The removal of free radicals and metal ions is essential for maintaining normal physiological function in humans (43, 44). Excessive levels of these reactive species can transfer electrons and oxidize key biomacromolecules-including proteins, DNA, and lipids-ultimately leading to oxidative damage and cellular dysfunction (45, 46). In the present study, QPHs produced using different enzymes displayed distinct antioxidant activities, with alcalase-treated hydrolysates consistently showing the strongest performance across all assays (47). This enhanced activity may be partly associated with the higher degree of hydrolysis and the predominance of smaller peptide fragments generated by alcalase treatment, which can increase the accessibility of reactive functional groups involved in redox reactions (40).

Similar observations have been reported for alcalase hydrolysates of mung bean and adzuki bean proteins, which exhibited superior ferric reducing antioxidant power compared with their native proteins at concentrations of 0.1–2 mg/ml (48). Furthermore, soybean protein hydrolysates (49) and defatted black cumin protein hydrolysates (50) also demonstrated strong antioxidant activities. It should be acknowledged that the present study did not incorporate peptide sequencing, activity-guided fractionation, or targeted analyses of residue enrichment. Accordingly, any associations discussed between antioxidant activity and particular amino acid residues or peptide motifs are proposed within the context of general trends reported for protein hydrolysate systems, rather than as definitive mechanistic interpretations derived directly from the current dataset. The bulk amino acid composition data (Table 1) offer a general compositional profile; however, they do not capture peptide sequence information or residue positioning, both of which are recognized as critical determinants of antioxidant functionality.

### 4.3 Effects of hydrolysates on cellular antioxidant activity

Low cytotoxicity of QPHs ensures their safety and provides a necessary premise for further investigation of their protective roles against oxidative cellular injury. QPHs produced using different enzymatic treatments consistently exhibited low cytotoxicity across a wide concentration range, maintaining high cell viability and demonstrating good biocompatibility. Cellular protection against oxidative damage was evaluated using cell viability assays and measurements of intracellular antioxidant enzyme activities, which reflect the capacity of exogenous antioxidant agents to counteract free radical- and peroxide-induced injury. All QPHs significantly improved cell survival and activated endogenous antioxidant enzyme systems, with the alcalase hydrolysate showing the strongest cytoprotective effects.

The D-galactose-induced oxidative stress model is a well-established *in vitro* system for assessing intracellular oxidative injury (51). Analyses of SOD, GPx, CAT, and MDA provided a comprehensive evaluation of the regulatory effects of the hydrolysates on cellular redox homeostasis. QPHs at different concentrations markedly modulated these antioxidant enzyme activities, with the alcalase hydrolysate group exhibiting significantly higher SOD, GPx, and CAT activities compared with the model group, accompanied by a substantial reduction in MDA levels, indicating superior inhibition of lipid peroxidation, consistent with previous reports showing that protein hydrolysates can enhance intracellular antioxidant enzyme activities and attenuate oxidative injury in HepG2 cells (52). Overall, these findings clearly demonstrate the strong functional potential of QPHs.

It should be explicitly noted that intracellular ROS levels and GSH content were not directly measured in this study. Accordingly, the observed effects are interpreted as reflecting antioxidant-supporting capacity and cytoprotection, rather than direct evidence of ROS scavenging, mitochondrial regulation, or apoptosis modulation. Despite this limitation, the coordinated improvements in cell viability, antioxidant enzyme activities, and lipid peroxidation indices provide biologically meaningful evidence for the functional potential of QPHs.

## 5 Conclusions

This study demonstrates that enzymatic hydrolysis significantly modulates the structural and functional properties of quinoa protein, leading to enhanced antioxidant potential in a strongly enzyme-dependent manner. Hydrolysis disrupted noncovalent interactions, reduced surface hydrophobicity, and produced smaller, more flexible QPHs, which collectively improved reducing power and free radical scavenging capacity, with alcalase-derived hydrolysates showing the most prominent activities and highest cytoprotection against D-galactose-induced oxidative damage. These effects are likely related to the release of antioxidant amino acid-rich fragments such as those containing methionine, glutamic acid, and aspartic acid. While the findings highlight the promising potential of quinoa protein hydrolysates-particularly those produced by alcalase-as natural antioxidants for functional food applications, limitations remain. Future research should therefore isolate key bioactive fragments, assess their bioavailability and mechanisms of action, and validate their efficacy in animal models and food systems to support practical applications.

## Data Availability

The original contributions presented in the study are included in the article/supplementary material, further inquiries can be directed to the corresponding author.

## References

[1] Park JH Lee SI Kwon WS Cho S Kim IH. Health benefits of co-supplementing mealworm protein hydrolysate and cranberry fruit extract. Ital J Food Sci. (2023) 35:1–9. doi: 10.15586/ijfs.v35i1.2264

[2] Yang C Zhu X Zhang Z Yang F Wei Y Zhang Z . Heat treatment of quinoa (*Chenopodium quinoa* Willd.) albumin: effect on structural, functional, and in vitro digestion properties. Front Nutr. (2022) 9:1010617. doi: 10.3389/fnut.2022.1010617 36185662 PMC9520662

[3] Nowak V Du J Charrondiere UR. Assessment of the nutritional composition of quinoa (*Chenopodium quinoa* Willd.). Food Chem. (2016) 193:47–54. doi: 10.1016/j.foodchem.2015.02.111 26433286

[4] Yang C Liu W Zhu X Zhang X Wei Y Huang J . Ultrasound-assisted enzymatic digestion for efficient extraction of proteins from quinoa. LWT-Food Sci Technol. (2024) 194:115784. doi: 10.1016/j.lwt.2024.115784

[5] Shen Z-J Xu S-X Huang Q-Y Li Z-Y Xu Y-D Lin C-S . TMT proteomics analysis of a pseudocereal crop, quinoa (*Chenopodium quinoa* Willd.), during seed maturation. Front Plant Sci. (2022) 13:975073. doi: 10.3389/fpls.2022.975073 36426144 PMC9678934

[6] Rafik S Rahmani M Rodriguez JP Andam S Ezzariai A El Gharous M . How does mechanical pearling affect quinoa nutrients and saponin contents? Plants. (2021) 10:1133. doi: 10.3390/plants10061133 34204858 PMC8230041

[7] Tabatabaei I Alseekh S Shahid M Leniak E Wagner M Mahmoudi H . The diversity of quinoa morphological traits and seed metabolic composition. Sci Data. (2022) 9:323. doi: 10.1038/s41597-022-01399-y 35725573 PMC9209433

[8] Woiciechowski AL Karp SG Sobral K de Carvalho JC Letti LAJ Soccol VT . Pretreatment strategies to enhance value addition of agro-industrial wastes. In:Brar SK Dhillon GS Soccol CR, editors. Biotransformation of Waste Biomass into High Value Biochemicals. New York, NY: Springer New York (2014). p. 29–49. doi: 10.1007/978-1-4614-8005-1_2

[9] Zheng Z Wei X Shang T Huang Y Hu C Zhang R. Bioconversion of duck blood cell: process optimization of hydrolytic conditions and peptide hydrolysate characterization. BMC Biotechnol. (2018) 18:67. doi: 10.1186/s12896-018-0475-5 30342496 PMC6196028

[10] Wani IA Sogi DS Shivhare US Gill BS. Physico-chemical and functional properties of native and hydrolyzed kidney bean (*Phaseolus vulgaris* L.) protein isolates. Food Res Int. (2015) 76:11–8. doi: 10.1016/j.foodres.2014.08.027

[11] Zu X Huang Y Zhao Y Xiong G Liao T Li H. Peptide extraction from silver carp (*Hypophthalmichthys molitrix*) scales via enzymatic hydrolysis and membrane filtration. Ital J Food Sci. (2023) 35:44–53. doi: 10.15586/ijfs.v35i2.2248

[12] Xie J Du M Shen M Wu T Lin L. Physico-chemical properties, antioxidant activities and angiotensin-I converting enzyme inhibitory of protein hydrolysates from Mung bean (*Vigna radiate*). Food Chem. (2019) 270:243–50. doi: 10.1016/j.foodchem.2018.07.103 30174041

[13] do Evangelho JA Vanier NL Pinto VZ Berrios JJD Dias ARG Zavareze ER. Black bean (*Phaseolus vulgaris* L) protein hydrolysates: physicochemical and functional properties. Food Chem. (2017) 214:460–7. doi: 10.1016/j.foodchem.2016.07.046 27507499

[14] Mansinhbhai CH Sakure A Liu Z Maurya R Das S Basaiawmoit B . Anti-inflammatory, ACE inhibitory, antioxidative activities and release of novel antihypertensive and antioxidative peptides from whey protein hydrolysate with molecular interactions. J Am Nutr Assoc. (2023) 42:371–85. doi: 10.1080/07315724.2022.2052201 35584265

[15] Chi C-F Wang B Deng Y-Y Wang Y-M Deng S-G Ma J-Y. Isolation and characterization of three antioxidant pentapeptides from protein hydrolysate of monkfish (*Lophius litulon*) muscle. Food Res Int. (2014) 55:222–8. doi: 10.1016/j.foodres.2013.11.018

[16] Sabeena Farvin KH Andersen LL Otte J Nielsen HH Jessen F Jacobsen C. Antioxidant activity of cod (*Gadus morhua*) protein hydrolysates: fractionation and characterisation of peptide fractions. Food Chem. (2016) 204:409. doi: 10.1016/j.foodchem.2016.02.145 26988519

[17] Yahia S Benomar S Dehiba F Allaoui A Guillen N Rodriguez-Yoldi MJ . Hypocholesterolaemic and antioxidant efficiency of chickpea (*Cicer arietinum*) protein hydrolysates depend on its degree of hydrolysis in cholesterol-fed rat. Nutr Food Sci. (2017) 47:254–69. doi: 10.1108/NFS-04-2016-0046

[18] Hunsakul K Laokuldilok T Sakdatorn V Klangpetch W Brennan CS Utama-ang N. Optimization of enzymatic hydrolysis by alcalase and flavourzyme to enhance the antioxidant properties of jasmine rice bran protein hydrolysate. Sci Rep. (2022) 12:12582. doi: 10.1038/s41598-022-16821-z 35869265 PMC9307646

[19] Mirzapour-Kouhdasht A McClements DJ Taghizadeh MS Niazi A Garcia-Vaquero M. Strategies for oral delivery of bioactive peptides with focus on debittering and masking. NPJ Sci Food. (2023) 7:22. doi: 10.1038/s41538-023-00198-y 37231034 PMC10212938

[20] Abbasi S Moslehishad M Salami M. Antioxidant and alpha-glucosidase enzyme inhibitory properties of hydrolyzed protein and bioactive peptides of quinoa. Int J Biol Macromol. (2022) 213:602–9. doi: 10.1016/j.ijbiomac.2022.05.189 35659938

[21] Tocmo R Balagiannis D Quinn E Finnegan D Collins M Loscher CE. Protein hydrolysates from quinoa (*Chenopodium quinoa* Willd.) modulate macrophage polarization and the expression of surface antigen molecules. Sustain Food Proteins. (2024) 2:282–96. doi: 10.1002/sfp2.1042

[22] Li R Wang Q Shen Y Li M Sun L. Integrated extraction, structural characterization, and activity assessment of squid pen protein hydrolysates and β-chitin with different protease hydrolysis. Int J Biol Macromol. (2024) 262:130069. doi: 10.1016/j.ijbiomac.2024.130069 38340918

[23] López-Moreno M Jiménez-Moreno E Márquez Gallego A Vera Pasamontes G Uranga Ocio JA Garcés-Rimón M . Red quinoa hydrolysates with antioxidant properties improve cardiovascular health in spontaneously hypertensive rats. Antioxidants. (2023) 12:1291. doi: 10.3390/antiox12061291 37372021 PMC10295081

[24] Du X Jing H Wang L Huang X Wang X Wang H. Characterization of structure, physicochemical properties, and hypoglycemic activity of goat milk whey protein hydrolysate processed with different proteases. LWT-Food Sci Technol. (2022) 159:113257. doi: 10.1016/j.lwt.2022.113257

[25] Nielsen PM Petersen D Dambmann C. Improved method for determining food protein degree of hydrolysis. J Food Sci. (2001) 66:642–6. doi: 10.1111/j.1365-2621.2001.tb04614.x

[26] Xu Y Yang Y Ma C Bian X Liu X Wang Y . Characterization of the structure, antioxidant activity and hypoglycemic activity of soy (*Glycine max* L.) protein hydrolysates. Food Res Int. (2023) 173:113473. doi: 10.1016/j.foodres.2023.113473 37803796

[27] Chelh I Gatellier P Sante-Lhoutellier V. Technical note: a simplified procedure for myofibril hydrophobicity determination. Meat Sci. (2006) 74:681–3. doi: 10.1016/j.meatsci.2006.05.019 22063223

[28] Feng X Tjia JYY Zhou Y Liu Q Fu C Yang H. Effects of tocopherol nanoemulsion addition on fish sausage properties and fatty acid oxidation. LWT-Food Sci Technol. (2020) 118:108737. doi: 10.1016/j.lwt.2019.108737

[29] Machado S Costa ASG Pimentel FB Oliveira MBPP Alves RC. A study on the protein fraction of coffee silverskin: protein/non-protein nitrogen and free and total amino acid profiles. Food Chem. (2020) 326:126940. doi: 10.1016/j.foodchem.2020.126940 32413751

[30] Repo-Carrasco-Valencia R Hellström J Pihlava J-M Pihlava J-M. Flavonoids and other phenolic compounds in Andean indigenous grains: quinoa (*Chenopodium quinoa*), kañiwa (*Chenopodium pallidicaule*) and kiwicha (*Amaranthus caudatus*). Food Chem. (2010) 120:128–33. doi: 10.1016/j.foodchem.2009.09.087

[31] Shao Y Hu Z Yu Y Mou R Zhu Z Beta T. Phenolic acids, anthocyanins, proanthocyanidins, antioxidant activity, minerals and their correlations in non-pigmented, red, and black rice. Food Chem. (2018) 239:733–41. doi: 10.1016/j.foodchem.2017.07.009 28873629

[32] Pang Y Ahmed S Xu Y Beta T Zhu Z Shao Y . Bound phenolic compounds and antioxidant properties of whole grain and bran of white, red and black rice. Food Chem. (2018) 240:212–21. doi: 10.1016/j.foodchem.2017.07.095 28946264

[33] You L Zhao M Cui C Zhao H Yang B. Effect of degree of hydrolysis on the antioxidant activity of loach (*Misgurnus anguillicaudatus*) protein hydrolysates. Innov Food Sci Emerg Technol. (2009) 10:235–40. doi: 10.1016/j.ifset.2008.08.007

[34] Wen C Zhang J Feng Y Duan Y Ma H Zhang H. Purification and identification of novel antioxidant peptides from watermelon seed protein hydrolysates and their cytoprotective effects on H_2_O_2_-induced oxidative stress. Food Chem. (2020) 327:127059. doi: 10.1016/j.foodchem.2020.127059 32447138

[35] Cui H Roy D Li S Loo TS Guo Q Ye A. Recovery and characterisation of quinoa protein via neutral to alkaline extraction: protein profiles and heat-induced aggregation behaviour. Curr Res Food Sci. (2025) 11:101214. doi: 10.1016/j.crfs.2025.101214 41114401 PMC12529351

[36] Lyu S Chen M-R Wang Y Zhang D Zhao S Liu J . Foaming properties of egg white proteins improved by enzymatic hydrolysis: the changes in structure and physicochemical properties. Food Hydrocoll. (2023) 141:108681. doi: 10.1016/j.foodhyd.2023.108681

[37] Wang J Liu J John A Jiang Y Zhu H Yang B . Structure identification of walnut peptides and evaluation of cellular antioxidant activity. Food Chem. (2022) 388:132943. doi: 10.1016/j.foodchem.2022.132943 35436638

[38] Liu F-F Li Y-Q Wang C-Y Liang Y Zhao X-Z He J-X . Physicochemical, functional and antioxidant properties of mung bean protein enzymatic hydrolysates. Food Chem. (2022) 393:133397. doi: 10.1016/j.foodchem.2022.133397 35679704

[39] Campos MRS Espinosa-García L Chel-Guerrero L Betancur-Ancona DA. Effect of enzymatic hydrolysis on solubility, hydrophobicity, and *in vivo* digestibility in cowpea (*Vigna unguiculata*). Int J Food Prop. (2012) 15:770–80. doi: 10.1080/10942912.2010.501469

[40] Correa JL Zapata JE Hernández-Ledesma B. Impact of alcalase hydrolysis and simulated gastrointestinal digestion on the release of bioactive peptides from *Erythrina edulis* (chachafruto) proteins. Int J Mol Sci. (2024) 25:9290. doi: 10.3390/ijms25179290 39273238 PMC11394852

[41] Korkmaz F Mutlu C. Safflower protein hydrolysates: physicochemical, functional properties and antioxidant activities. Food Sci Nutr. (2025) 13:e70258. doi: 10.1002/fsn3.70258 40357341 PMC12066243

[42] Chen Y Zheng Z Ai Z Zhang Y Tan CP Liu Y. Exploring the antioxidant and structural properties of black bean protein hydrolysate and its peptide fractions. Front Nutr. (2022) 9:884537. doi: 10.3389/fnut.2022.884537 35734370 PMC9207475

[43] Wang Q Wang Y Huang M Hayat K Kurtz NC Wu X . Ultrasound-assisted alkaline proteinase extraction enhances the yield of pecan protein and modifies its functional properties. Ultrason Sonochem. (2021) 80:105789. doi: 10.1016/j.ultsonch.2021.105789 34689068 PMC8551211

[44] Zahra N Mushtaq I Rehman A Fatima S Khalid A Sarwar A . In-vivo and *In-silico* analysis of the anti-inflammatory, antipyretic, and analgesic activities of methanolic fruit extracts of *Carica papaya. Ital J Food Sci*. (2024) 36:120–35. doi: 10.15586/ijfs.v36i4.2742

[45] Kim J-S Lee Y-S. Antioxidant activity of Maillard reaction products derived from aqueous glucose/glycine, diglycine, and triglycine model systems as a function of heating time. Food Chem. (2009) 116:227–32. doi: 10.1016/j.foodchem.2009.02.038

[46] Mohamadi N Meraghni M Merdaci F Necib A El Arbi M Elhadef K . Investigation and quantification of the potential antioxidant, inflammatory, and antibacterial bioactive molecules of the extracts of Algerian black and green table olive brine. Qual Assur Saf Crops Foods. (2023) 15:92–106. doi: 10.15586/qas.v15i1.1250

[47] De Carvalho Oliveira L Martinez-Villaluenga C Frias J Elena Cartea M Francisco M Cristianini M . High pressure-assisted enzymatic hydrolysis potentiates the production of quinoa protein hydrolysates with antioxidant and ACE-inhibitory activities. Food Chem. (2024) 447:138887. doi: 10.1016/j.foodchem.2024.138887 38492299

[48] Karami Z Butkinaree C Yingchutrakul Y Simanon N Duangmal K. Comparative study on structural, biological and functional activities of hydrolysates from Adzuki bean (*Vigna angularis*) and mung bean (*Vigna radiata*) protein concentrates using Alcalase and Flavourzyme. Food Res Int. (2022) 161:111797. doi: 10.1016/j.foodres.2022.111797 36192943

[49] Zhang Q Yu Z Zhao W. Identification and action mechanism of novel antioxidative peptides from copra meal protein. LWT-Food Sci Technol. (2023) 188:115425. doi: 10.1016/j.lwt.2023.115425

[50] Shahi ZKM Sayyed-Alangi SZ Najafian L. Effects of enzyme type and process time on hydrolysis degree, electrophoresis bands and antioxidant properties of hydrolyzed proteins derived from defatted *Bunium persicum* Bioss. press cake. Heliyon. (2020) 6:e03365. doi: 10.1016/j.heliyon.2020.e03365 32072055 PMC7015986

[51] Sun J Zhou C Cao J He J Sun Y Dang Y . Purification and characterization of novel antioxidative peptides from duck liver protein hydrolysate as well as their cytoprotection against oxidative stress in HepG2 cells. Front Nutr. (2022) 9:848289. doi: 10.3389/fnut.2022.848289 35369059 PMC8965237

[52] Rajapakse N Mendis E Jung W-K Je J-Y Kim S-K. Purification of a radical scavenging peptide from fermented mussel sauce and its antioxidant properties. Food Res Int. (2005) 38:175–82. doi: 10.1016/j.foodres.2004.10.002

